# Serum Fatty Acids Are Associated with a Higher Risk of Ischemic Stroke

**DOI:** 10.3390/nu15030585

**Published:** 2023-01-22

**Authors:** Sebastian Andone, Lénárd Farczádi, Silvia Imre, Zoltan Bajko, Anca Moțățăianu, Smaranda Maier, Laura Bărcuțean, Rodica Bălașa

**Affiliations:** 1 Ist Neurology Clinic, Emergency Clinical County Hospital Targu Mures, 540136 Targu Mures, Romania; 2 Department of Neurology, ‘George Emil Palade’ University of Medicine, Pharmacy, Science, and Technology of Targu Mures, 540142 Targu Mures, Romania; 3 Doctoral School, ‘George Emil Palade’ University of Medicine, Pharmacy, Science, and Technology of Targu Mures, 540142 Targu Mures, Romania; 4 Center for Advanced Medical and Pharmaceutical Research, ‘George Emil Palade’ University of Medicine, Pharmacy, Science, and Technology of Targu Mures, 540142 Targu Mures, Romania; 5 Department of Analytical Chemistry and Drug Analysis, Faculty of Pharmacy, ‘George Emil Palade’ University of Medicine, Pharmacy, Science, and Technology of Targu Mures, 540142 Targu Mures, Romania

**Keywords:** ischemic stroke, fatty acids, risk factors, arachidonic acid, eicosapentaenoic acid, docosahexaenoic acid, omega-3, omega-6

## Abstract

Stroke prevention, a significant public-health concern, begins with recognizing and addressing risk factors. Interventions targeted at modifiable risk factors can effectively prevent ischemic stroke, while Omega-3 fatty acids have been shown to improve stroke outcomes. Our study aimed to investigate the relationship between ischemic-stroke risk factors and fatty acids using a prospective observational study with 274 patients. We collected clinical data on risk factors and measured fatty-acid levels using high-performance liquid chromatography coupled with mass spectrometry. We found that several risk factors, including age, sex, smoking, atrial fibrillation, dyslipidemia, and previous stroke history, had a direct relationship with fatty acids. Of these, smoking had the most significant impact, negatively impacting levels of docosahexaenoic and eicosapentaenoic acid. Conversely, dyslipidemia and atrial fibrillation positively correlated with fatty acids, particularly in female patients and those with recurrent strokes. Age was found to directly correlate with other risk factors and variations in fatty-acid ratios. The stroke rate was higher in males than females before the age of 70, but this trend reversed. Our findings suggest that better management of risk factors, particularly modifiable lifestyle factors, could improve fatty-acid profiles and the balance of Omega-3 and Omega-6 in patients with ischemic stroke.

## 1. Introduction

Stroke represents a public-health problem with increasing costs for the healthcare system. Identifying stroke risk factors represented one of the leading research focuses on understanding stroke physiopathology and the impact of the modifiable risk factors.

When assessing the burden of a specific disease, several factors have been taken into account, such as epidemiological indicators (mortality rates, total number of specific disease cases quantified as incidence or prevalence), the use of inpatient and outpatient services, and the impact of the disease over the economic loss (absences, inability to work, use of medical facilities) [1].

The burden of managing stroke is high, with associated extended hospital care and continued health expenditures after discharge; therefore, the efforts for prevention and motivating healthy lifestyles are well-justified [2].

The prevention of ischemic stroke starts with identifying risk factors such as hypertension, dyslipidemia, systemic atherosclerosis, diabetes mellitus, cardiovascular disease, and stroke history [3]. To reduce the worldwide stroke burden, a proposed strategy included individual interventions over risk factors such as smoking, alcohol consumption, and dyslipidemia and populational interventions for the primary prevention of associated comorbidities that could represent a stroke risk factor [4].

Many ischemic-stroke risk factors exist, modifiable and non-modifiable, short- and long-term outcomes, age-dependent (young patients have different risk factors compared to old patients), and dependent on the ischemic-stroke subtype [5]. Primary and secondary prevention objectives include smoking cessation, diet change, weight control, and increased physical activity, all of which are directly connected to the lipid profile of patients [6].

Fatty acids are carbon chains with a methyl group at one end and a carboxyl group at the other. They have an essential function in the metabolism, serving as energy storage, a component of cellular membranes, and genetic regulators [7]. Additionally, certain fatty acids can regulate gene expression or transcription activity, impacting metabolic activity in a broader sense by controlling gene expression and other processes, for example, the insulin metabolic pathway [8]. Additionally, these fatty acids possess powerful anti-inflammatory abilities, as prostaglandin-3, among other prostaglandins, employ them as precursors in their synthesis [9]. Furthermore, fatty acids are believed to be beneficial for children’s visual, behavioral and cognitive capabilities, such as learning and attention [10].

Structurally, monounsaturated fatty acids (MUFA) possess one double bond. In contrast, polyunsaturated fatty acids (PUFA) have multiple double bonds, with the first one located at the third and fourth carbon atoms (N-3 or Omega 3) or between the sixth and seventh carbon atoms (N-6 or Omega 6) [7].

**Figure 1:**
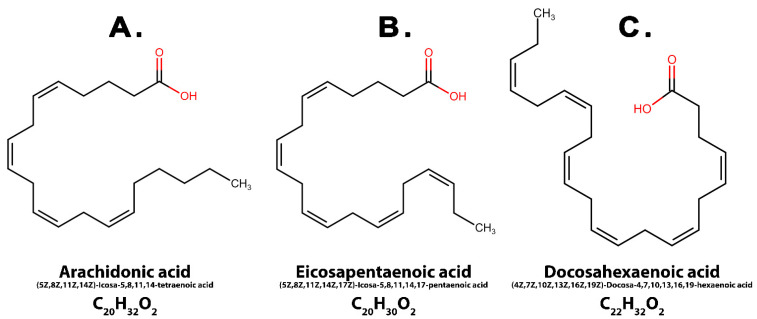
Fatty-acids’ chemical structures.

Arachidonic acid (AA) is an Omega-6 long-chain fatty acid derived from linoleic acid (LA) with a 20-carbon atom chain containing four unsaturated double bonds (20:4). The human body cannot synthesize this fatty acid, and its intake has to be ensured from food sources such as eggs, fish, soy, sunflower oil, poultry and peanuts [11] (Figure 1A). 

Eicosapentaenoic acid (EPA) is a long-chain Omega-3 fatty acid consisting of 20 carbon atoms and possessing five unsaturated double bonds (20:5). It can be consumed from sources such as fish oil and algae oil [12] (Figure 1B).

Docosahexaenoic acid (DHA) is another Omega-3 fatty acid containing a 22-carbon chain with six double bounds. It can be found in seafood and fish meat such as sardine, salmon, and herring [12] (Figure 1C).

**Figure 2:**
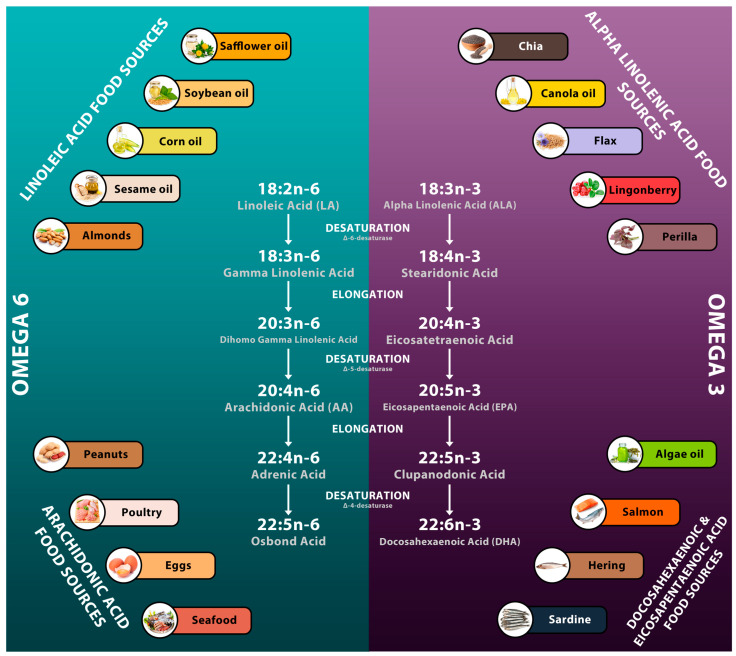
Omega-3 and Omega-6—sources and pathway.

Alpha-linolenic acid (ALA) is the precursor to both EPA and DHA and is derived from various sources, including chia, canola, flax, lingonberry, and perilla. For ALA to be converted into EPA and DHA, it must first be desaturated by the ∆-6-desaturase enzyme, forming stearidonic acid. An elongation follows this to a 20-atom carbon chain resulting in eicosatetraenoic acid, desaturated using the ∆-5-desaturase enzyme to create EPA. An additional elongation to a 22-atom carbon chain, known as clupanodonic acid, is necessary before another desaturation by the ∆-4-desaturase enzyme yields DHA [13] (Figure 2).

The composition of fatty acids in the diet has been found to affect lipidic directly as well as glycemic metabolism, aggregation, and erythrocyte deformation, consequently affecting blood pressure values. Eicosapentaenoic acid (EPA) and docosahexaenoic acid (DHA) have been observed to influence the production of eicosanoids with vasoactive aldosterone properties and modulate nitric oxide secretion from the endothelium, contributing to a reduction in blood pressure [14,15,16]. Additionally, DHA and EPA have been identified to reduce the levels of prostaglandin E and tumor growth factors [17,18,19], while playing a role in stabilizing atherosclerotic plaques [20,21]. The relationship between macronutrients and dietary replacement of polyunsaturated fatty acids (PUFAs) merits further research, which should include an evaluation of patients’ dietary patterns [22,23]. 

It has been established that there is a direct connection between the risk of an ischemic stroke and an individual’s metabolic and physiological status. Furthermore, the serum concentration levels of the Omega 6:Omega 3 ratio is a significant indicator of physiological dysfunction and the onset of insulin resistance in stroke survivors [24].

Studies have also demonstrated a correlation between the classic lipid profile and the age of onset in those who have experienced a stroke. Furthermore, it has been suggested that patients of middle age with elevated amounts of Omega-6 PUFAs are more likely to experience a lacunar or atherosclerotic stroke, although very limited data exists for elderly patients [25].

Additionally, evidence has suggested that serum concentration values are more reliable markers of changes in the fatty-acid profile when compared to the percentage of weight (composition) [26].

A buildup of fatty acids can result in oxidative phosphorylation and the formation of reactive chemicals through inflammatory pathways. The concentrations of fatty acids in the serum and cerebrospinal fluid can help predict the short-term prognosis of a stroke at the time of onset and serve as an independent indicator of stroke severity [27].

Cigarette smoking is a risk factor directly correlated to the fatty-acid profile, particularly Omega-3 fatty acids [28]. Its connection to fatty acids is believed to be due to the peroxidation of PUFAs caused by oxidative stress [29]. Smoking is a major contributor to atherosclerosis and is also linked to other unhealthy behavior, such as a decreased intake of Omega-3 food or supplements [30].

Gender has also been demonstrated to impact fatty-acid concentration, as men have been found to have lower serum levels of AA and DHA than women [31]. The Framingham Study previously reported a lower incidence rate of ischemic stroke in women, so the role of fatty acids in this connection should be further explored [32].

Scientific studies have established a strong correlation between the additional intake of PUFAs, specifically Omega-3 fatty acids, and their beneficial effects on cardiovascular and cerebrovascular health [33].

The purpose of our study is to establish the correlations between risk factors and fatty-acids profile in order to create a baseline of reference for future studies which may address specific risk factors prevention actions, or which may focus more on the links between serum fatty acids and ischemic-stroke risk factors and the mechanism involved in these.

## 2. Results

### 2.1. Study Population Analysis 

We included a total number of 274 patients consisting of 143 (52.2%) male and 131 (47.8%) female patients. The mean age was 70.53 +/− 12.63 years; the youngest patient was 25 years old, while the oldest was 98 years old.

Serum values of the fatty acids and their ratios are summarized in Table 1.

**Figure 3:**
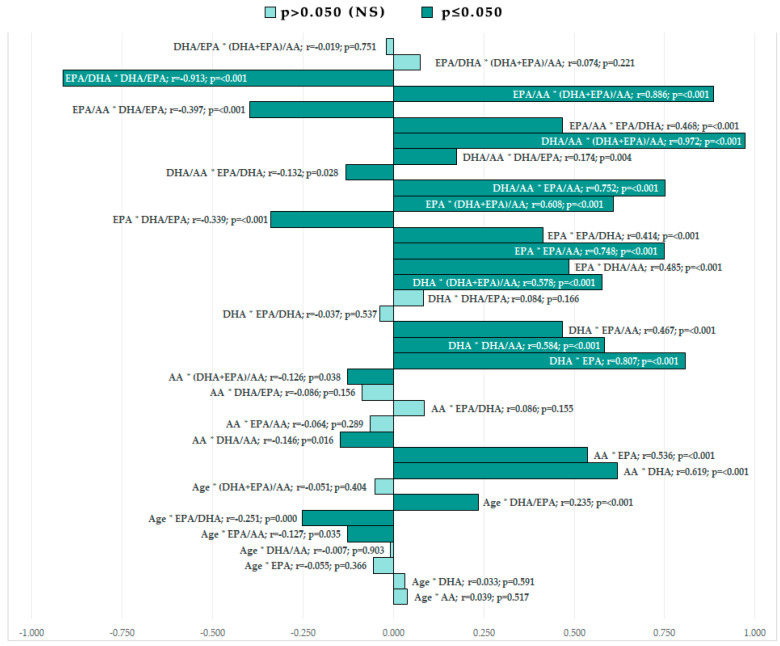
Pearson correlation graphical representation between age, fatty-acid serum values, and fatty-acid ratios. (* = correlation between).

The Pearson correlation between fatty-acid levels, their ratios, and the patient’s age can be found in Figure 3. We must mention that the serum levels of the fatty acids positively correlate, and the age positively correlates with the DHA/EPA ratio but is negative with EPA/DHA without having any other correlations with the studied parameters.

From the metabolic and cardiovascular risk factors, 144 (52.6%) of the patients had dyslipidemia, 74 (27%) had diabetes mellitus, 253 (92.3%) had hypertension, 11 (4%) had peripheral obliterative arteriopathy, and 20 (7.3%) had chronic kidney disease. 

**Figure 4:**
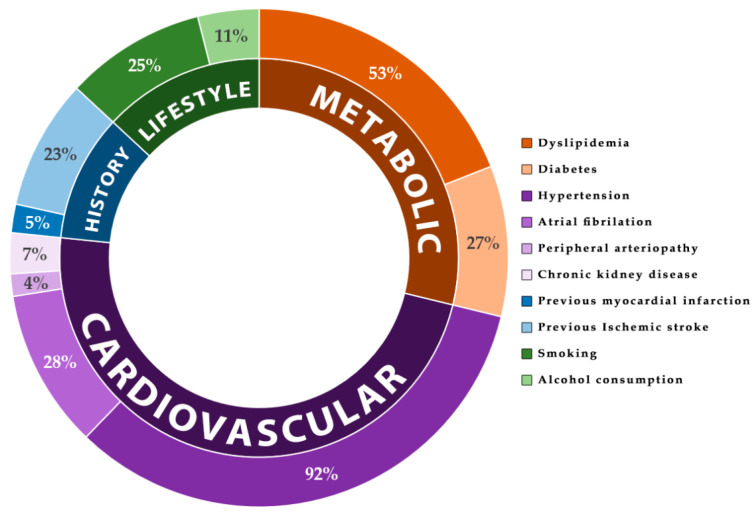
Risk-factor frequency distribution.

From the cardiovascular events’ history, 14 (5.1%) patients had a previous myocardial infarction, and 64 (23.4%) had a previous ischemic stroke. Regarding the lifestyle risk factors, 69 (25.2%) of the patients were smokers, and 30 (10.9%) were chronic alcohol consumers. All these data can be found in Table 2 and Figure 4.

Between the studied risk factors, we observed statistically significant associations between sex and hypertension (*p* = 0.006), sex and atrial fibrillation (*p* = 0.005), sex and smoking (*p* < 0.001), and sex and chronic alcohol consumption (*p* < 0.001) (Table 3).

Besides this, we also observed associations between smoking and hypertension (*p* = 0.019), smoking and atrial fibrillation (*p* < 0.001), smoking and chronic kidney disease (*p* = 0.031), and smoking and chronic alcohol consumption (*p* < 0.001); dyslipidemia and diabetes mellitus (*p* = 0.030), dyslipidemia and atrial fibrillation (*p* = 0.011); ad chronic kidney disease with atrial fibrillation (*p* = 0.010). No other significant associations between risk factors were discovered (Table 4).

The mean age of the male patients was 67.66 +/− 13.31 years, while the mean age of female patients was 73.66 +/− 11.06 (*p* < 0.001). We also observed statistically significant differences between the EPA/DHA ratio (*p* = 0.008) and DHA/EPA ratio (*p* = 0.002) between male and female patients. 

**Figure 5:**
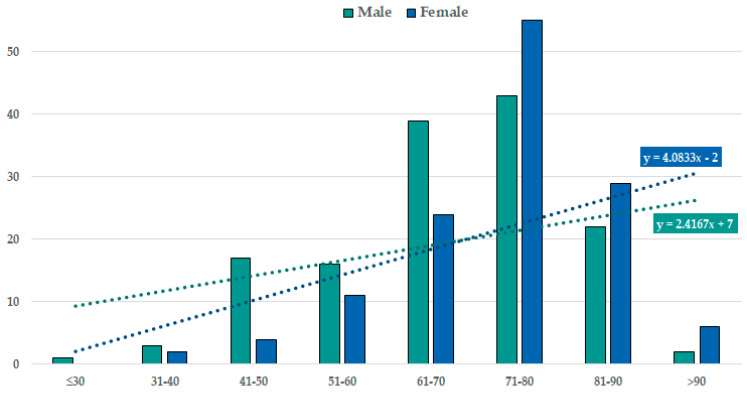
Age-group distribution based on gender.

The patients’ age distribution based on sex can be seen in Figure 5. We applied a linear trendline model for the two subgroups and obtained two equations to describe ischemic-stroke incidence based on age group (y = 2.4167x + 7 for male patients and y = 4.0833x − 2 for female patients). We obtained an intersection value for the two trendlines at an approximate value of 69.5 years old, a point which represents the age at which the stroke incidence in male patients and female patients is almost equal. Therefore, the female stroke incidence was lower than the male under this value, but higher after.

Regarding dyslipidemia, we observed statistically significant differences in all three fatty acids: AA (*p* = 0.033), DHA (*p* = 0.003), and EPA (*p* = 0.006). In the patients grouped by hypertension, atrial fibrillation, and chronic kidney disease, we observed extremely significant differences in mean age (*p* < 0.001).

The smoker patients had a mean age of 62.62 +/− 11.69 years compared to the non-smokers, who had a mean age of 72.30 +/− 11.81 years (*p* < 0.001). Between the two groups, we also observed differences in the serum levels of DHA (*p* = 0.021) as well as DHA/AA ratio (*p* = 0.009), EPA/DHA ratio (*p* = 0.012), DHA/EPA ratio (*p* = 0.023) and (DHA + EPA)/AA ratio (*p* = 0.029). All these data are summarized in Table 5.

We divided the patients into subgroups based on each of the 10 risk factors studied; however, we obtained relevant data only for the gender subgroups, dyslipidemia subgroups, and stroke-history subgroups.

Dyslipidemia and gender were the only risk factors with almost equal percentages between the subgroups, thus offering better insight into the differences, as the subgroups were homogenous. Although the stroke-history subgroups offered less homogenous data, it was the only prior-history risk factor which had relevant difference results.

All other subgroups offered no statistically significant results and were not included in the tables.

### 2.2. Sex-Groups Subanalysis

In the male patient group, the only statistically significant differences regarding risk factors were related to the mean age. In the male patients with hypertension, the mean age was 69.52 +/− 11.92 compared to the male patients without hypertension, with a mean age of 53.94 +/− 15.35 (*p* < 0.001). Statistically significant differences in the mean age were also observed in the subgroups of male patients grouped by atrial fibrillation (*p* < 0.001), chronic kidney disease (*p* = 0.002), and smoking (*p* < 0.001).

In the female patients with dyslipidemia, we observed a serum level of DHA of 1407.49 +/− 979.20 ng/mL compared with the female patients without dyslipidemia who had a value of 1079.29 +/− 520.85 ng/mL (*p* = 0.033). The female patients that had atrial fibrillation, when compared with female patients without atrial fibrillation, had statistically significant differences regarding mean age (*p* = 0.005), AA serum values (*p* = 0.039), DHA serum values (*p* = 0.050) and EPA serum values (*p* = 0.038).

Besides this, the only differences observed in female patients were in the mean age of the subgroups grouped by chronic kidney disease (*p* = 0.034) and smoking (*p* < 0.001).

All these data can be found in Table 6.

### 2.3. Dyslipidemia-Groups Subanalysis

In the patients with dyslipidemia, we observed an extremely significant difference between the mean age of the male and female patients (*p* < 0.001). Grouped based on atrial fibrillation, the patients with dyslipidemia had statistically significant differences regarding mean age (*p* < 0.001), DHA serum values (*p* = 0.050), and EPA serum values (*p* = 0.013). In addition, dyslipidemic patients grouped by smoking had statistically significant differences regarding mean age (*p* < 0.001) and EPA/DHA ratio (*p* = 0.036). In the dyslipidemic patients who suffered a previous stroke compared with those who had no previous history, we observed differences regarding DHA serum values (*p* = 0.005), EPA serum values (*p* = 0.008) but also regarding DHA/AA ratio (*p* = 0.005), EPA/AA ratio (*p* = 0.006) and (DHA+EPA)/AA ratio (*p* = 0.003).

In the non-dyslipidemic patient group, we observed differences between male and female patients regarding mean age (*p* = 0.037), EPA/DHA ratio (*p* = 0.006), and DHA/EPA ratio (*p* = 0.004). We also observed differences in the mean age of non-dyslipidemic patients grouped by hypertension (*p* < 0.001), atrial fibrillation (*p* < 0.001), chronic kidney disease (*p* = 0.002), smoking (*p* < 0.001), and chronic alcohol consumption (*p* = 0.024).

Besides this, in the non-dyslipidemic patients grouped by chronic kidney disease, we also observed differences in the EPA/DHA ratio (*p* = 0.013) and DHA/EPA ratio (*p* = 0.008), respectively, grouped by smoking, where there is a difference of the DHA/AA ratio (*p* = 0.045). All these data can be found in Table 7.

### 2.4. Stroke-History-Group Subanalysis

In patients with recurrent stroke with hypertension, the mean age was 72.97 +/− 11.46 years, while the recurrent stroke patients without hypertension had a mean age of 39.33 +/− 14.01, an extremely statistically significant difference (*p* < 0.001).

Other observed differences were between mean age in the groups grouped by sex (*p* = 0.035), atrial fibrillation (*p* = 0.002), and smoking (*p* = 0.001). 

In the recurrent-stroke patients’ groups based on dyslipidemia, we found statistically significant differences between the serum values of AA (*p* = 0.022), DHA (*p* = 0.004), EPA (*p* = 0.012) and between their ratios DHA/AA (*p* = 0.022), EPA/AA (*p* = 0.029), (DHA + EPA)/AA (*p* = 0.017).

Other differences in the recurrent-stroke group were found when grouped by atrial fibrillation regarding EPA serum values (*p* = 0.040); when grouped by smoking regarding DHA/AA ratio (*p* = 0.048), EPA/DHA ratio (*p* = 0.030), DHA/EPA ratio (*p* = 0.029); and when grouped by chronic alcohol consumption regarding AA serum values (*p* = 0.022), and DHA/AA ratio (*p* = 0.050).

In the patients’ group with no previous-stroke history, we observed differences regarding mean age when grouped by sex (*p* = 0.001), hypertension (*p* < 0.001), atrial fibrillation (*p* < 0.001), chronic kidney disease (*p* = 0.001), smoking (*p* < 0.001) and chronic alcohol consumption (*p* = 0.015).

Between the female and male patients that had no stroke history, we observed differences regarding DHA serum values (*p* = 0.034), DHA/AA ratio (*p* = 0.037), EPA/DHA ratio (*p* = 0.004), and DHA/AA ratio (*p* = 0.004).

Other differences observed were when grouped by smoking, regarding DHA serum values (*p* = 0.028), respectively, and when grouped by diabetes regarding DHA/AA ratio (*p* = 0.050) and (DHA + EPA)/AA (*p* = 0.045).

All the data can be found summarized in Table 8.

## 3. Discussion

Our study aimed to observe the relationships between the risk factors in ischemic-stroke patients and the fatty-acid profile containing AA, DHA, EPA, and their ratios using high-performance liquid chromatography coupled with mass spectrometry in order to determine if we can influence the fatty-acids profile by taking a direct action over a specific risk factor. As a summary of the evidence, our results show that some risk factors, such as dyslipidemia, atrial fibrillation, and smoking, have solid connections with the levels of fatty acids and their ratios. On the other hand is the history of myocardial infarction and peripheral obliterative arteriopathy, which have no apparent relationship with the fatty-acid profile in ischemic-stroke patients. Gender and age also seem to play a role in the link between risk factors and fatty acids, as older patients, especially female patients, have a more significant correlation between several risk factors, such as dyslipidemia and atrial fibrillation, as opposed to male patients. All these findings will be discussed more extensively later.

Regarding fatty-acids profile, we observed a positive correlation between them. Regarding bioavailability, the serum value of AA was the highest among these three, followed by DHA and EPA.

Hypertension was the most common risk factor, and was present in almost all included patients. The rest of the risk factors, such as diabetes mellitus, atrial fibrillation, smoking, and previous-stroke history, represented around a quarter of the total number of patients. The rarest associated risk factors were, in descending order of frequency: chronic alcohol consumption, chronic kidney disease, previous myocardial infarction history, and peripheral obliterative arteriopathy. 

From all the collected data, we observed that arteriopathy and a history of myocardial infarction had no connection with the fatty-acid panel. Even more, these two risk factors had no correlations or associations with the other risk factors for ischemic stroke.

### 3.1. Age and Sex Distribution

Regarding the studied population, we observed a somewhat equal distribution between male and female patients and between other parameters, such as dyslipidemia. 

Female patients were older than male patients; a reason for this could be the male-gender predisposition to developing a ischemic stroke at a younger age.

Caso et al. proved, in a group of 1136 patients, from which female patients represented 46%, that female patients develop ischemic stroke at a higher age than male patients. This is according to our results. In the same study, the authors suggest that this age difference is based on the increased life span of the female compared to the male [34].

Gibson et al. assign this difference to the dependent and independent hormonal mechanisms, but supports the same finding: that females develop ischemic stroke at an older age than males [32].

A similar age difference between males and females was pointed out through a meta-analysis, by Gargano et al., of first-time ischemic-stroke patients [35].

Patients with hypertension, atrial fibrillation, or chronic kidney disease were older than patients who did not present these associated risk factors. The exact relation could be observed in both genders, but only for atrial fibrillation and chronic kidney disease. Hypertension is associated with a higher mean age only in the male group but not in the female patient group. Male patients are more predisposed to hypertension than female patients as age increases. On the other hand, smoker ischemic-stroke patients were younger than non-smokers. The same could be observed separately for female and male patients, respectively. This could be explained by the predisposition of younger patients to have associated lifestyle risk factors, or with older patients being more predisposed to giving up these vices.

In female patients with atrial fibrillation, higher values of all fatty acids were observed compared to female patients without atrial fibrillation.

### 3.2. Lifestyle Risk Factors—Smoking and Fatty Acids

The relationship between fatty acids and smoking has been extensively studied; one of the first studies on this subject was published by Santos et al. in 1984, which showed that middle-age male smokers present an increased value of fatty acids, including EPA, but a decrease in other fatty acids, such as AA, in comparison with control non-smoker patients [30,36]. Unfortunately, these patients’ DHA value was not studied. We also have to mention that the inclusion criteria comprised only patients with no other associated risk factors, to assess this relationship independently [37]. This could explain partially why in our study, in smoker non-dyslipidemic patients, DHA/AA ratio is lower in comparison with non-smoker patients, as an increase in AA plasmatic value could influence this ratio, even though we could not demonstrate a significant increase in serum AA for this subgroup.

Another study, by Simon et al., showed an inverse relationship between smoking with the serum values of DHA and AA [36]. This inverse relationship of smoking with DHA value was also present in our study population but is most frequent in smoker patients with an initial stroke. Besides this, the ratios between DHA and AA and the rest of the fatty acids support these findings. 

Baldassare et al. emphasized this observation, again proving a decrease in AA and DHA in smoker patients compared to non-smokers [38]. Of course, this relationship between smoking and fatty acids can also be explained by dietary intake.

To support this, Scaglia et al. tried to study the relationship between diets enriched in Omega-3 and smoking. Not only did they prove that smokers had lower levels of DHA and EPA, but they also observed that these patients tend to eat smaller amounts of food enriched in Omega-3 fatty acids, especially fish, than non-smokers [30].

In smoker patients, on the other hand, DHA had lower values than non-smokers, with a direct influence on a decrease in DHA/AA, DHA/EPA, and (DHA + EPA)/AA ratio in smokers in comparison to non-smoker patients. The only ratio which was higher in smoker patients was EPA/DHA. This suggests that DHA could be a stratification marker, as lower values will be associated with smoking in stroke patients. Any decrease in DHA/AA, DHA/EPA, and (DHA + EPA)/AA ratio at the same time as a decrease in EPA/DHA could also be used for the same role.

### 3.3. Dyslipidemia and Fatty Acids

We observed higher values of all monitored fatty acids in dyslipidemic patients than in non-dyslipidemic patients. The same could not be demonstrated in the male patient subgroup, but female patients with dyslipidemia presented with a higher value of DHA than non-dyslipidemic female patients. 

Mori et al. also stated that fatty acids correlate with dyslipidemia in ischemic stroke patients and consider that targeted therapies regarding fatty acids could improve lipid-profile serum values [39].

Although, for the entire studied population, we could not find statistically significant differences between the mean age of dyslipidemic patients, as well as for non-dyslipidemic patients, when we performed a different comparison in the dyslipidemic-patient subgroup as well as in the non-dyslipidemic-patient subgroup, we observed a significantly lower age of men compared to female patients. This is most likely influenced by the fact that male patients had a much lower age than female patients, as we previously mentioned.

We must also mention that we noticed a direct association between dyslipidemia and atrial fibrillation. As in the general population, we observed higher mean ages in non-dyslipidemic patients with associated risk factors such as atrial fibrillation, chronic kidney disease, and hypertension. The same was observed in dyslipidemic patients, but only for atrial fibrillation. 

Non-dyslipidemic patients with both lifestyle risk factors had a lower mean age than patients without these risk factors. This could be observed only for smoking but not chronic alcohol consumption in dyslipidemic patients. 

We observed higher DHA and EPA values in dyslipidemic patients with atrial fibrillation than those without atrial fibrillation. On the other hand, this modification could not be observed in non-dyslipidemic patients. In dyslipidemic patients with a previous ischemic stroke, higher values of DHA and EPA and DHA/AA, EPA/AA, and (DHA + EPA)/AA were observed. In non-dyslipidemic patients, we could not observe any modifications regarding previous ischemic stroke history.

Tanaka et al. showed that a diet with highly purified EPA reduces the risk of recurrent stroke in dyslipidemic patients [40].

### 3.4. Recurrent Ischemic Stroke and Fatty Acids 

In patients with recurrent storke, higher values of all fatty acids and DHA/AA, EPA/AA, and (DHA + EPA)/AA ratios were observed in patients with dyslipidemia compared to non-dyslipidemic patients. On the other hand, no difference was noted in non-recurrent ischemic-stroke patients. 

Patients with recurrent stroke and atrial fibrillation had a higher EPA value than patients without atrial fibrillation, without an influence on the fatty-acid ratios. These differences were not observed in patients without recurrent stroke. In smoker patients with non-recurrent stroke, we observed a lower value of DHA compared to non-smoker patients with non-recurrent stroke. This was not observed in patients with recurrent stroke, but we observed lower values of DHA/AA and DHA/EPA as well as EPA/DHA in smoker patients compared to non-smokers for patients with a recurrent stroke. 

Nelson et al. showed that a decreased EPA/AA ratio is associated with increased cardiovascular events [41]. We also observed that this ratio was higher in both dyslipidemic patients with a previous stroke than in those with no previous history of stroke, proving that EPA/AA ratio can be a marker of future events.

### 3.5. Literature Additional Data and Future Prospects

Veno et al. conducted a retrospective study that raised the issue of limited data surrounding the dietary intake of linoleic acid and its potential association with a decreased risk of ischemic stroke. Furthermore, their findings suggested that the level of linoleic acid in adipose tissue was inversely related to the risk of ischemic stroke, particularly in small vessel disease, and that it may have a protective effect against atherothrombotic stroke [42].

Simon et al. conducted a study of approximately 200 middle-aged men and found that serum alpha-linolenic acid was inversely associated with the risk of stroke, whereas the stearic acid serum values were directly associated with the risk of ischemic stroke. Our study did not compare these fatty acids but noted similar correlations of studied fatty acids with the risk of developing stroke and general risk factors. Additionally, we evaluated the impact these fatty acids had on patient prognosis, which is an important factor for future research [43].

Mori et al., in their study regarding the association between fatty-acid serum values at admission and the age of onset in ischemic stroke, concluded the importance of assessing in research studies the type and quantities of dietary lipid intake in the days prior to developing ischemic stroke. The correlation between the concentrations and the ratios of fatty acids and the dietary intake of triglycerides from meat, fish, oils, and vegetables supported this. Our group of patients did not provide information regarding their initial dietary intake. However, we consider that we could raise awareness regarding this specific issue through the dietary recommendations provided [44].

Adding further PUFAs to the method should be possible and might be part of future research. However, for this study, the research team decided it would be relevant to focus on and measure the PUFAs for which the method had already been developed and validated.

Taking into consideration that several studies showed the impact which fatty acids have on the clinical prognosis of ischemic-stroke patients by improving functional outcome, mortality, as well as their prognostic value in the long term, the attempt to influence the serum values of fatty acids and their ratios could be achieved with better control over risk factors and also over the elimination of certain risk factors, especially lifestyle risk factors such as smoking and chronic alcohol consumption [41,45,46].

## 4. Limitations

Although our study is observational, one of the limitations or a future extension could represent a comparison between fatty-acid profiles in patients with ischemic stroke with a control (matched) group.

A further limitation of the study was the profiling of fatty acids. A more thorough evaluation of other fatty acids, such as alpha-linolenic acid, myristic, palmitic, and stearic acid, may have delivered more insight into the correlation between fatty acids and ischemic stroke.

During the development of the LC-MS method, in addition to AA, EPA, and DHA, other omega PUFA were also considered to be included, especially alfa linolenic acid and gamma-linolenic acid; however, due to time and financial limitations, the bioanalytical research team had to limit the development to only the three final PUFAs.

For this stage of the study, the research team wanted to find correlations for plasma PUFA as a first step, and, based on these results, will focus a future study on determining the omega index from red-blood-cell membranes, a value which is less influenced by immediate dietary intake and biological variability, allowing for an integral and retrograde evaluation of omega PUFA. The method available to the research team was validated for measuring free PUFA in plasma and PUFA from red-blood-cell membranes incorporated into triglycerides. Due to being only semi-quantitative and involving highly increased costs and sample preparation times, PUFA measurement from RBC membranes was decided to be the focus of a future study of the research team, during which the plasma omega PUFA levels will likely be normalized to total triglyceride and total lipid levels, compared to membrane values, in order to determine any relevant correlations to ischemic stroke risk.

## 5. Materials and Methods

We performed a prospective observational study covering ischemic-stroke patients admitted to 1st Neurology Clinic, Emergency Clinical County Hospital, Târgu Mureș over six months (January 2022–June 2022).

We included all admitted patients diagnosed with acute ischemic stroke but excluded any patients with hemorrhagic stroke, transient ischemic attack, or stroke-mimic pathology.

Collected data were included in a digital research platform which included clinical data (demographic, related to associated risk factors or associated comorbidities), paraclinical data, and personal data from the files of the patients admitted after obtaining written informed consent by the patients (or legal guardians/family members). The study was conducted in conformity with Helsinki Declaration and was previously approved by the Ethics Committee of Emergency Clinical County Hospital (no. 28763/13.11.2018).

Peripheral blood samples were collected using EDTA vacutainers with a gel separator to determine fatty acids profile—AA, DHA, EPA, in the first 24 h from admission. After the probes were priorly centrifuged at 3500 rpm for 15 min, we extracted plasma and deposited it into cryotubes at −40 °C.

In order to quantify serum values of AA, DHA, and EPA, we used an analytical method consisting of high-performance liquid chromatography (HPLC) coupled with mass spectrometry (MS). We used an internal standard isotopically labeled arachidonic-d11 acid. We, furthermore, analyzed the samples using reversed-phase liquid chromatography (LC). Additionally, we detected with specific transition using the mass spectrometer’s MRM MS/MS detection after negative electrospray ionization (ESI-). The samples and the calibration standards were freshly prepared, and we used the following concentration range of standard calibration solutions: AA (2.5, 12.5, 25, 62.5, 125, 250 µg/mL), DHA, and EPA (50, 250, 500, 1250, 2500 ng/mL).

For the preparation of the samples, we added in an Eppendorf tube 200 µL of patient plasma, 100 µL of internal standard, and 500 µL of acetonitrile, whereas, for the preparation of calibration standard solution, we added in Eppendorf tubes 200 µL of work solution, 100 µL of internal standard 1 µg/mL arachidonic-d11 acid in acetonitrile, and 500 µL of acetonitrile.

For the detection of the analytes and internal standard, we based our method on specific fragmentation patterns after negative ion spray ionization: the sum of ions *m*/*z* 234.94 and m/z 259.27 formed from the fragmentation of parent ion *m*/*z* 303.25 at a collision energy of −16 V for AA; the sum of ions *m*/*z* 203.19 and *m*/*z* 257.25 formed from the fragmentation of parent ion *m*/*z* 301.15 at a collision energy of −16 V for EPA; the sum of ions *m*/*z* 229.22, *m*/*z* 249.21 and *m*/*z* 283.26 formed from the fragmentation of parent ion *m*/*z* 327.25 at a collision energy of −13V for DHA; and ion *m*/*z* 270.35 formed from the fragmentation of parent ion *m*/*z* 314.26 at a collision energy of −16V for the internal standard.

For mass spectrometric detection, we used a Q-TOF 4600 Sciex system, and the following chromatographic conditions were applied using a Perkin–Elmer Flexar 10 UHPLC system: chromatographic column Kinetex XB-C18 with dimensions 3.0 × 100mm, 2.6 um; mobile phase 15%—ammonium formate 10 mM and 85%—acetonitrile; flowrate 0.400 mL/min; sample runtime 6 min; column thermostat temperature 25 °C; sample thermostat temperature 20 °C; and injection volume 5 µL.

For calibration, we applied a linear calibration model composed of 6 levels for AA and 5 for DHA and EPA using 1/y2 weighing.

For both statistical analysis and graphical figures, we used software/online applications such as www.chemspace.com (accessed on 01 August 2022) online application and Adobe Photoshop CS4, IBM SPSS Statistics v26, and Microsoft Excel 2019.

The statistical analyses consisted of an assessment of parametric variables (ANOVA test), describing the data as continuous (mean, standard deviation (SD), median, min/max), depending on the distribution. Quantitative variables were correlated using the Pearson correlation coefficient (rho), set at alpha = 0.05. *p*-value was set at ≤0.05 for significance. In order to assess the correlation between the distributions of the categorical variables, we used contingency tables and the Chi2 test.

## 6. Conclusions

Ischemic stroke presents a variety of risk factors, including metabolic and cardiovascular, regarding patients’ history and lifestyle risk factors. The role that fatty acids play in the physiopathology of ischemic stroke can be explained by their relationships with specific ischemic stroke risk factors.

Risk factors such as dyslipidemia, atrial fibrillation, and smoking are strongly connected to fatty acids. On the other hand, peripheral obliterative arteriopathy and a history of previous myocardial infarction are not impactful risk factors for fatty acids.

Another classic risk factor for ischemic stroke, age, showed a direct relationship with the other risk factors and the different ratios of fatty acids. In the case of gender, however, male patients had younger ages than female patients. Although the incidence of stroke was higher in males than females before the seventh decade, this is reversed from that point forward.

Smoking appears to be the highest influencing risk factor over fatty acids, being inversely proportional to the serum value of DHA and DHA ratios with the rest of the fatty acids. Dyslipidemia and atrial fibrillation positively correlate with fatty acids, especially in female patients. A positive correlation between the same risk factors and the lipidic profile has also been established in patients with recurrent stroke, regardless of sex.

Better control of risk factors, especially the elimination of modifiable lifestyle risk factors such as smoking, could influence the fatty-acids profile and the ratios between Omega-3 and Omega-6 fractions in patients with ischemic stroke.

## Figures and Tables

**Table 1:** Descriptive statistics.

Variable (n = 274)	Mean ± Standard Deviation
Age (years)	70.53 ± 12.63
AA serum values (µg/mL)	250.27 ± 121.04
DHA serum values (ng/mL)	1178.17 ± 798.40
EPA serum values (ng/mL)	425.45 ± 366.84
DHA/AA ratio (‰)	4.86 ± 2.21
EPA/AA ratio (‰)	1.73 ± 1.12
EPA/DHA ratio	0.36 ± 0.13
DHA/EPA ratio	3.05 ± 0.95
(DHA+EPA)/AA ratio (‰)	6.60 ± 3.14

**Table 2:** Frequency table.

	Male	Female
Sex	143 (52.2%)	131 (47.8%)
**Metabolic risk factors**		
	**YES**	**NO**
Dyslipidemia	144 (52.6%)	130 (47.4%)
Diabetes	74 (27.0%)	299 (73.0%)
**Cardiovascular risk factors**		
	**YES**	**NO**
Hypertension	253 (92.3%)	21 (7.7%)
Atrial fibrillation	78 (28.5%)	196 (71.5%)
Peripheral obliterative arteriopathy	11 (4.0%)	263 (96.0%)
Chronic kidney disease	20 (7.3%)	254 (92.7%)
**History risk factors**		
	**YES**	**NO**
Previous myocardial infarct	14 (5.1%)	260 (94.9%)
Previous ischemic stroke	64 (23.4%)	210 (76.6%)
**Lifestyle risk factors**		
	**YES**	**NO**
Smoking	69 (25.2%)	205 (74.8%)
Alcohol consumption	30 (10.9%)	244 (89.1%)

**Table 3:** Crosstabs between sex and risk factors.

Sex		Male	Female	*p*-Value
Dyslipidemia	Yes	69	75	0.147
	No	74	56	
Diabetes Mellitus	Yes	33	41	0.136
	No	110	90	
Hypertension	Yes	126	127	0.006
	No	17	4	
Atrial fibrillation	Yes	30	48	0.005
	No	113	83	
Peripheral obliterative arteriopathy	Yes	9	2	0.063
	No	134	129	
Chronic kidney disease	Yes	8	12	0.353
	No	135	119	
Previous myocardial infarct	Yes	10	4	0.174
	No	133	127	
Previous ischemic stroke	Yes	32	32	0.775
	No	111	99	
Smoking	Yes	54	15	<0.001
	No	89	116	
Alcohol consumption	Yes	27	3	<0.001
	No	116	128	

**Table 4:** Crosstabs between risk factors.

Smoking		Yes	No	*p*-Value
Hypertension	Yes	59	194	0.019
	No	10	11	
Atrial fibrillation	Yes	6	72	<0.001
	No	63	133	
Chronic kidney disease	Yes	1	19	0.031
	No	68	186	
Alcohol consumption	Yes	20	10	<0.001
	No	49	195	
**Dyslipidemia**		**Yes**	**No**	
Diabetes Mellitus	Yes	47	27	0.030
	No	97	103	
Atrial fibrillation	Yes	31	47	0.011
	No	113	83	
**Chronic kidney disease**		**Yes**	**No**	
Atrial fibrillation	Yes	11	9	0.010
	No	67	187	

**Table 5:** Study population ANOVA analysis.

	Sex		*p*-Value
	**Male**	**Female**	
Age (years)	67.66 ± 13.31	73.66 ± 11.06	<0.001
EPA/DHA	0.38 ± 0.12	0.34 ± 0.12	0.008
DHA/EPA	2.87 ± 0.88	3.23 ± 0.99	0.002
	**Dyslipidemia**		
	**Yes**	**No**	
AA (µg/mL)	265.03 ± 125.42	233.91 ± 125.42	0.033
EPA/DHA	0.37 ± 0.14	0.35 ± 0.10	0.003
DHA/EPA	2.99 ± 0.94	3.10 ± 0.96	0.006
	**Hypertension**		
	**Yes**	**No**	
Age (years)	71.66 ± 11.48	57.00 ± 17.51	<0.001
	**Atrial fibrillation**		
	**Yes**	**No**	
Age (years)	77.14 ± 10.05	67.90 ± 12.61	<0.001
	**Chronic kidney disease**		
	**Yes**	**No**	
Age (years)	80.60 ± 9.52	69.74 ± 12.51	<0.001
	**Smoking**		
	**Yes**	**No**	
Age (years)	62.62 ± 11.69	73.20 ± 11.81	<0.001
DHA (ng/mL)	985.96 ± 513.91	1242.86 ± 865.04	0.021
DHA/AA (‰)	4.26 ± 1.96	5.06 ± 2.25	0.009
EPA/DHA	0.39 ± 0.14	0.35 ± 0.11	0.012
DHA/EPA	2.82 ± 0.95	3.12 ± 0.94	0.023
(DHA + EPA)/AA (‰)	5.88 ± 2.67	6.83 ± 3.24	0.029

**Table 6:** Gender groups ANOVA subanalysis.

Male Patients Group			
	Hypertension		
	Yes	No	
Age (years)	69.52 ± 11.92	53.94 ± 15.35	<0.001
	Atrial fibrillation		
	Yes	No	
Age (years)	77.07 ± 9.38	65.17 ± 13.11	<0.001
	Chronic kidney disease		
	Yes	No	
Age (years)	81.38 ± 10.30	66.85 ± 13.05	0.002
	Smoking		
	Yes	No	
Age (years)	62.35 ± 11.95	70.89 ± 13.12	<0.001
**Female Patients group**			
	Dyslipidemia		
	Yes	No	
DHA (ng/mL)	1407.49 ± 979.20	1097.29 ± 520.85	0.033
	Atrial fibrillation		
	Yes	No	
Age (years)	77.19 ± 10.55	71.63 ± 10.90	0.005
AA (µg/mL)	289.69 ± 169.03	240.01 ± 103.53	0.039
DHA (ng/mL)	1460.57 ± 1040.08	1167.50 ± 658.02	0.050
EPA (ng/mL)	533.01 ± 635.31	386.93 ± 265.05	0.038
	Chronic kidney disease		
	Yes	No	
Age (years)	80.08 ± 9.40	73.02 ± 11.04	0.034
	Smoking		
	Yes	No	
Age (years)	63.60 ± 11.02	74.97 ± 10.42	<0.001

**Table 7:** Dyslipidemia groups ANOVA subanalysis.

Dyslipidemic Patients Group			
	Sex		
	Male	Female	
Age (years)	65.46 ± 11.19	72.87 ± 10.83	<0.001
	Atrial fibrillation		
	Yes	No	
Age (years)	76.52 ± 9.75	67.35 ± 11.28	<0.001
DHA (ng/mL)	1612.41 ± 1251.19	1229.76 ± 860.98	0.050
EPA (ng/mL)	658.53 ± 769.37	435.07 ± 293.44	0.013
	Previous ischemic stroke		
	Yes	No	
DHA (ng/mL)	1736.58 ± 1536.59	1195.69 ± 704.58	0.005
EPA (ng/mL)	669.93 ± 767.22	431.94 ± 292.46	0.008
DHA/AA (‰)	6.08 ± 3.73	4.71 ± 1.80	0.005
EPA/AA (‰)	2.42 ± 2.34	1.68 ± 0.81	0.006
(DHA+EPA)/AA (‰)	8.50 ± 5.79	6.39 ± 2.45	0.003
	Smoking		
	Yes	No	
Age (years)	63.32 ± 10.73	71.39 ± 11.17	<0.001
EPA/DHA	0.41 ± 0.16	0.35 ± 0.12	0.036
**Non-dyslipidemic Patients Group**			
	Sex		
	Male	Female	
Age (years)	69.72 ± 14.81	74.73 ± 11.38	0.037
EPA/DHA	0.37 ± 0.10	0.32 ± 0.10	0.006
DHA/EPA	2.89 ± 0.88	3.37 ± 1.01	0.004
	Hypertension		
	Yes	No	
Age (years)	73.94 ± 11.65	54.79 ± 16.95	<0.001
	Atrial fibrillation		
	Yes	No	
Age (years)	77.55 ± 10.33	68.66 ± 14.25	<0.001
	Chronic kidney disease		
	Yes	No	
Age (years)	83.08 ± 9.17	70.63 ± 13.49	0.002
EPA/DHA	0.28 ± 0.07	0.36 ± 0.10	0.013
DHA/EPA	3.77 ± 0.98	3.02 ± 0.94	0.008
	Smoking		
	Yes	No	
Age (years)	61.81 ± 12.83	75.16 ± 12.24	<0.001
DHA/AA (‰)	4.09 ± 1.99	4.89 ± 1.91	0.045
	Alcohol		
	Yes	No	
Age (years)	64.14 ± 17.51	72.81 ± 12.85	0.024

**Table 8:** Stroke-history groups ANOVA subanalysis.

Recurrent Stroke Patients Group			
	Sex		
	Male	Female	
Age (years)	67.84 ± 15.72	74.94 ± 9.90	0.035
	Dyslipidemia		
	Yes	No	
AA (µg/mL)	287.86 ± 167.80	210.07 ± 86.34	0.022
DHA (ng/mL)	1736.58 ± 153.59	909.83 ± 381.72	0.004
EPA (ng/mL)	669.93 ± 767.22	315.68 ± 160.50	0.012
DHA/AA (‰)	6.08 ± 3.73	4.45 ± 1.37	0.022
EPA/AA (‰)	2.42 ± 2.34	1.49 ± 0.43	0.029
(DHA + EPA)/AA (‰)	8.50 ± 5.79	5.94 ± 1.63	0.017
	Hypertension		
	Yes	No	
Age (years)	72.97 ± 11.46	39.33 ± 14.01	<0.001
	Atrial fibrillation		
	Yes	No	
Age (years)	69.37 ± 7.64	68.02 ± 14.09	0.002
EPA (ng/mL)	711.81 ± 926.20	392.46 ± 289.41	0.040
	Smoking		
	Yes	No	
Age (years)	62.06 ± 14.56	74.77 ± 11.51	0.001
DHA/AA (‰)	4.06 ± 1.84	5.67 ± 3.07	0.048
EPA/DHA	0.44 ± 0.18	0.35 ± 0.13	0.030
DHA/EPA	2.55 ± 0.81	3.18 ± 1.04	0.029
	Alcohol		
	Yes	No	
AA (µg/mL)	381.20 ± 221.25	236.44 ± 123.71	0.022
DHA/AA (‰)	2.82 ± 1.42	5.44 ± 2.88	0.050
**Non-recurrent Stroke Patients Group**			
	Sex		
	Male	Female	
Age (years)	67.61 ± 12.61	73.25 ± 11.43	0.001
DHA (ng/mL)	1049.23 ± 512.55	1237.31 ± 751.82	0.034
DHA/AA (‰)	4.48 ± 1.90	5.04 ± 1.97	0.037
EPA/DHA	0.38 ± 0.12	0.33 ± 0.10	0.004
DHA/EPA	2.87 ± 0.91	3.25 ± 0.92	0.004
	Diabetes Mellitus		
	Yes	No	
DHA/AA (‰)	4.30 ± 1.56	4.90 ± 2.05	0.050
(DHA + EPA)/AA (‰)	5.81 ± 2.19	6.63 ± 2.76	0.045
	Hypertension		
	Yes	No	
Age (years)	71.24 ± 11.49	59.94 ± 16.53	<0.001
	Atrial fibrillation		
	Yes	No	
Age (years)	76.42 ± 10.67	67.87 ± 12.18	<0.001
	Chronic kidney disease		
	Yes	No	
Age (years)	81.14 ± 8.45	69.49 ± 12.25	0.001
	Smoking		
	Yes	No	
Age (years)	62.81 ± 10.74	72.73 ± 11.90	<0.001
DHA (ng/mL)	968.85 ± 390.58	1193.53 ± 697.52	0.028
	Alcohol		
	Yes	No	
Age (years)	64.64 ± 10.96	71.03 ± 12.38	0.015

## Data Availability

The data presented in this study are available on request from the corresponding author. The data are not publicly available due to institutional privacy restrictions.
